# Orofacial myofunctional therapy associated with the use of the stimulating palatal plate in children with trisomy 21: case studies

**DOI:** 10.1590/2317-1782/20232021231en

**Published:** 2023-09-01

**Authors:** Jéssica Ellen de Almeida Ferreira, Bruna Rezende Santos de Almeida, Tahyná Duda Deps, Henrique Pretti, Renata Maria Moreira Moraes Furlan

**Affiliations:** 1 Graduação em Fonoaudiologia, Faculdade de Medicina, Universidade Federal de Minas Gerais - UFMG - Belo Horizonte (MG), Brasil.; 2 Departamento de Fonoaudiologia, Faculdade de Medicina, Universidade Federal de Minas Gerais - UFMG - Belo Horizonte (MG), Brasil.; 3 Faculdade de Tecnologia do Ipê - FAIPE - Cuiabá (MT), Brasil.; 4 Departamento de Dentística Restauradora, Faculdade de Odontologia, Universidade Federal de Minas Gerais - UFMG - Belo Horizonte (MG), Brasil.

**Keywords:** Down Syndrome, Muscle Hypotonia, Rehabilitation, Myofunctional Therapy, Orthotic Devices

## Abstract

Individuals with trisomy 21 may have muscle hypotonia of the speech articulation organs, an enlarged protruding tongue positioned on the floor of the mouth, and a lack of lip closure. The stimulating palatal plate is an intraoral appliance that, associated with myofunctional therapy, aims to improve these children’s habitual lip and tongue posture. This study aimed to present the cases of four male children with trisomy 21, with a mean age of 6.7 and a standard deviation of 7.8 months, who used the stimulating palatal plate in association with myofunctional therapy. The children used the plate for 6 months and did exercises based on the orofacial regulation therapy, and their parents received instructions on feeding them and removing deleterious oral habits. In the first session and at the end of the treatment, each child’s face was video-recorded for 5 minutes at rest, and two researchers analyzed independently their habitual tongue and lip posture. Participants who began the treatment earlier and had the most severe postural changes had greater tongue and lip posture improvement.

## INTRODUCTION

Trisomy 21 (T21) is a chromosomal change characterized by a series of congenital conditions that interfere with motor and neurophysiological development, such as motor dysfunctions and muscle hypotonia^(1)^. Data from the Brazilian Ministry of Health^(2)^ indicate that one out of every 700 newborns in Brazil is diagnosed with T21, totaling about 270 thousand people. Individuals with T21 may have a smaller maxilla, midface hypoplasia, tongue protrusion, and lip closure difficulties^(3)^. These conditions directly impact functions such as mastication, swallowing, phonation, and breathing^(4)^.

In the 1970s, Argentine physician Castillo-Morales^(5)^ developed a neuromotor rehabilitation method for children with disabilities, consisting of Orofacial Regulation Therapy (ORT) with muscle stimulation exercises. He also proposed using an intraoral appliance named stimulating palatal plate (SPP) in combination with ORT.

SPP is an appliance produced by dentists based on the model of the child’s upper arch. It has been described in studies on the treatment of individuals with T21 to adjust the habitual tongue position and enable lip closure^(6,7)^. A longitudinal study followed up on 20 children with T21 using SPP associated with ORT for 4 years and reported that the treatment had a positive effect on their oral motor function, especially in their first year of life, highlighting improved tonus and lip closure^(8)^.

Few studies have addressed the benefits of using SPP associated with myofunctional therapy, and the time of treatment for muscle changes to take place has not been well-defined yet. Hence, this study reports the results on tongue and lip posture after using SPP associated with myofunctional therapy for 6 months in children with T21.

## PRESENTATION OF THE CLINICAL CASES

This prospective study approached four cases. The research was approved by the institution’s Research Ethics Committee (CAAE 37828920.1.0000.5149 - evaluation report: 4.381.966). The participants' parents/guardians signed an informed consent form, agreeing with the research and disclosure of its results.

Four children diagnosed with T21, all males, with a mean age of 6.7 months and a standard deviation of 7.8 months, participated in the research. None of them had any other associated syndrome, craniofacial malformation, or cardiac or respiratory disorder. They were recruited from among those referred for treatment at a public outreach program of the Federal University of Minas Gerais.

The children were assessed by a dentist and a speech-language-hearing therapist in the first session. The orofacial myofunctional assessment involved lip and tongue tonus^(9,10)^ and habitual posture^(11)^, the lingual frenulum^(10)^, the diet^(9)^, and oral habits^(10)^. All of them had decreased lip and tongue tonus and abnormal habitual posture, whereas their lingual frenulum was normal. The main orofacial myofunctional findings are shown in Chart 1.

**Chart 1:** Main findings of the children’s orofacial myofunctional assessment

**Participant (age)**	**Main findings of the myofunctional assessment**
Participant 1 (6 months)	**Lip and tongue tonus:** decreased.
	**Tongue posture:** on the floor of the mouth.
	**Lip posture**: sometimes open, sometimes closed.
	**Diet**: liquid food in a baby bottle and mashed fruits served on a small plastic spoon, without complaints. Breastfeeding up to 3 months old.
	**Deleterious sucking habits**: absent.
	**Teeth:** absent.
Participant 2 (1 month)	**Lip and tongue tonus:** decreased.
	**Tongue posture:** on the lower lip.
	**Lip posture:** open.
	**Diet:** Breastfeeding complemented with infant formula, without complaints.
	**Deleterious sucking habits**: absent.
	**Teeth**: absent.
Participant 3 (18 months)	**Lip and tongue tonus:** decreased.
	**Tongue posture:** intercalating between the tongue on the lower lip and inside the oral cavity but lowered on the floor of the mouth.
	**Lip posture:** open.
	**Diet:** solid, liquid, and pureed food, with occasional complaints of choking with solid foods.
	**Deleterious sucking habits:** absent.
	**Teeth:** present upper and lower central incisors.
Participant 4 (2 months)	**Lip and tongue tonus:** decreased.
	**Tongue posture:** on the lower lip.
	**Lip posture:** open.
	**Diet:** breastfeeding complemented with infant formula; complaints of choking.
	**Deleterious sucking habits:** pacifier.
	**Teeth:** absent.

**Figure 1:**
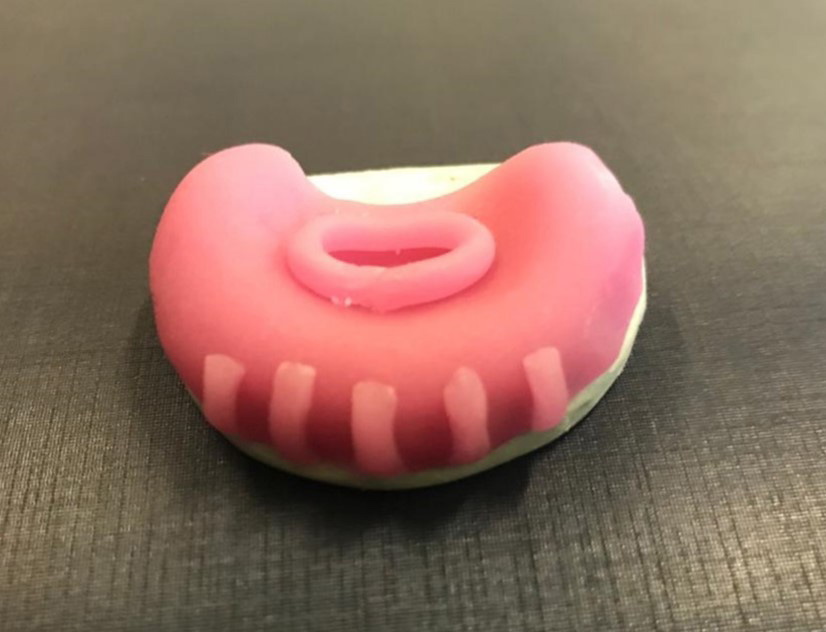
Stimulating palatal plate (SPP)

The dentist made a model of each child’s upper arch for the SPP, which was delivered the following week to their parents/guardians. An example of SPP is shown in Figure 1.

During the first session, the speech-language-hearing therapist made high-quality video recordings of each child’s face for 5 minutes, using a semiprofessional digital camera manufactured by Sony, model DSC-H50 (Sony^®^, Manaus, Brazil). They were positioned on a child safety seat or the parent/guardian’s lap, who were instructed not to interfere with the recordings. The children were not wearing SPP during the recordings. Appropriate toys for each age were used to distract the children, as the purpose was to pick up their habitual lip and tongue posture.

In the second session, which took place 1 week after the assessment, the participants received the SPP. The parents were instructed to insert in the child’s oral cavity four times a day for at least 30 minutes^(12)^ and learned how to proceed with SPP hygiene and not to have them wear it during meals or sleep^(5)^. Moreover, they were asked to use some therapeutic strategies every day to strengthen orofacial muscles, as described in Chart 2 ^(5)^. All these strategies were conducted by the lead speech-language-hearing researcher in the presence of the parents to train them, so they could repeat them at home. The strategies were also filmed, and the videos were made available to the families, along with a booklet with these explanations.

**Chart 2:** Exercises indicated in the treatment

**Exercise**	**Description**	**Repetition**
**1) Strengthening the masseter**	Sliding the fingertips of both hands from the angle of the mandible up toward the eyes.	10 times each movement
**2) Stretching the upper lip**	Sliding the tips of the index fingers and thumbs down from the center of the upper lip to the commissures.	10 times each movement
**3) Stretching the lower lip**	Sliding the tips of the index fingers and thumbs up from the center of the lower lip to the commissures.	10 times each movement
**4) Stimulating the lower nasal motor zone**	Pressing the index finger horizontally above the upper lip and vibrating it up and backward.	10 times each movement
**5) stimulating the lip motor zone**	Pressing the tips of the index fingers on the zygomaticus major muscle, simultaneously vibrating and pulling it.	10 times each movement
**6) Stimulating the chin motor zone**	Placing the index finger under the face and the thumb on the chin and making downward movements, simultaneously pressing and vibrating them.	10 times each movement
**7) Stimulating the tongue motor zone**	Pressing the thumb or index finger under the face, in the submandibular region of the neck, and vibrating intermittently.	10 times each movement
**8) Tongue vibration**	Pressing the index finger over the tongue and vibrating it intermittently. Then, perform mandible control.	20 times each movement
**9) Lifting the tip of the tongue**	Placing the index finger behind the lower gingiva (without touching the teeth) and the thumb in the submandibular region, then lifting the tip of the index finger to take the tongue near the upper teeth. Then, perform mandible control.	10 times each movement
**10) Tongue tapering**	Touching the lateral margins of the tongue backward with the index finger or a toothbrush.	10 times on each side of the tongue
**11) Lip vibration**	Flexing the finger joints to cup the hand, placing it over the child’s lips carefully not to hinder nose breathing. Then pressing the lips and face with a vacuum effect and vibrating upward.	20 times each movement
**12) Mandible control**	Placing the index finger on the chin, the middle finger below the mandible, and the thumb along the border of the mandible and resting the head on the arm.	Whenever the child needs alignment.

Source: Castillo-Morales^(5)^.

The third session took place 14 days after the second one, and the fourth session, after 2 months. These sessions aimed to reinforce the instructions on the therapeutic strategies to strengthen the orofacial muscles. The families were also instructed to remove deleterious oral habits and learned about the correct latch and position during breastfeeding (in cases 2 and 4) and how to offer food, position the child during meals, and use appropriate utensils (in cases 1 and 3). These sessions were carried out in person.

The families were free to contact the professionals via phone calls or teleconsultation to ask questions regarding SPP use and exercises whenever necessary. The number of sessions for each clinical case varied according to the need to make a new plate or answer questions the family had. Cases 1 and 3 had four in-person sessions and one teleconsultation session. Cases 2 and 4 had six in-person sessions and one teleconsultation session because their SPP had to be remade, as the children’s palates had grown - i.e., one extra session to make the new model and another one to deliver the new plate.

After 6 months of treatment, they were reassessed, and their faces were rerecorded in similar conditions as in the initial assessment. The children were not wearing SPP during the recordings.

Two researchers independently analyzed the videos frame by frame. In each frame, the child’s tongue posture was classified as I) inside the oral cavity (tongue behind the lower alveolar ridge or the lower incisors); II) between the alveolar ridges (tongue on the lower alveolar ridge and behind the lower lip); III) on the lower lip (tongue touching the lower lip); IV) severe protrusion in relation to the lower lip (protruded tongue on the lower lip, with its tip outside the anterior end of the lower lip)^(11)^. Lip posture was classified as I) closed (upper and lower lips fully in contact); II) parted (upper and lower lips in contact only near the corners of the mouth); III) open (no contact between upper and lower lips). The researchers counted the seconds in which the child remained in each classification of habitual lip and tongue posture. However, the moments when the child smiled or vocalized were not considered in the analysis. Data were compared between the assessment and reassessment after 6 months.

Two researchers analyzed the videos to increase data reliability. The agreement between them was verified with intraclass correlation coefficient. Each participant’s data were qualitatively analyzed. The intraclass correlation coefficient was 0.98 for participant 1; 0.95 for participant 2; 0.99 for participant 3; and 0.98 for participant 4 - which indicates an excellent interrater agreement.

Tables 1 and 2 show the time each participant remained in the various tongue and lip postures in the video recordings made at the beginning and end of the treatment.

**Table 1:** Time participants remained in each lip posture at the beginning and end of the treatment

Assessment moment	Participant	Age (months)	Lip posture					
			Closed		Parted		Open	
			Time (min)	%	Timed (min)	%	Time (min)	%
Beginning of the treatment	1	6	191	66.1	37	12.8	61	21.1
	2	1	1	0.4	2.5	0.9	269	98.7
	3	18	9.5	4.3	7.5	3.4	205	92.3
	4	2	8	3.3	7	2.9	226.5	93.8
After 6 months of treatment	1	12	37	16.7	78	35.3	106	48.0
	2	7	1	0.7	28.5	20.6	109	78.7
	3	24	10	5.7	19.5	11.1	146	83.2
	4	8	9.5	8.2	73	62.7	34	29.2

**Caption:** min = minutes.

**Table 2:** Time participants remained in each tongue posture at the beginning and end of the treatment

Assessment moment	Participant	Age (months)	**Tongue posture**							
			Inside the oral cavity		Between the alveolar ridges		On the lower lip		Severe protrusion	
			Time (min)	%	Time (min)	%	Time (min)	%	Time (min)	%
Beginning of the treatment	1	6	271.5	93.3	9.5	3.3	0.5	0.2	9.5	3.3
	2	1	0	0.0	14	5.5	218.5	86.5	20	7.9
	3	18	87	40.4	48	22.3	79	36.7	1.5	0.7
	4	2	14.5	6.1	7	2.9	186	77.7	32	13.4
End of the treatment	1	12	168	83.6	29.5	14.7	2.5	1.2	1	0.5
	2	7	7.5	4.2	68	38.3	102	57.5	0	0
	3	24	55.5	30	34	18.4	94.5	51.1	1	0.5
	4	8	36	30.8	53.5	45.7	24.5	20.9	3	2.6

**Caption:** min = minutes.

**Figure 2:**
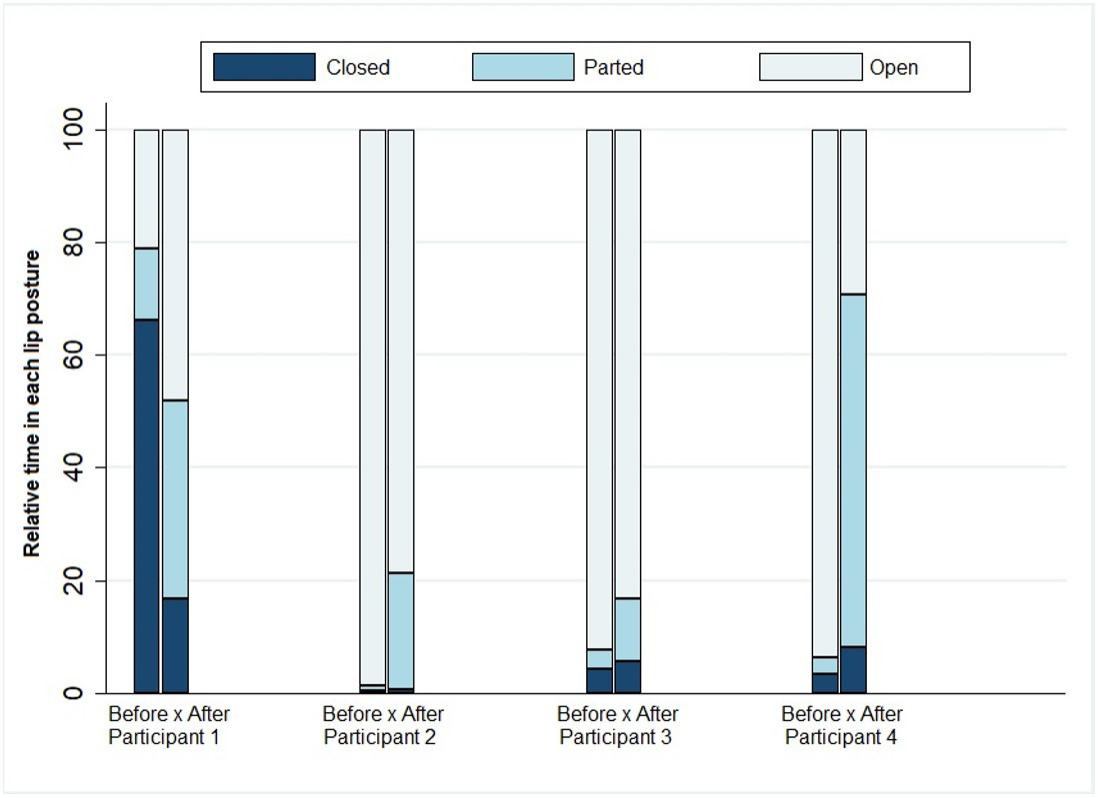
Percentage of the time participants remained in each lip posture at the beginning and end of the treatment in relation to video duration

**Figure 3:**
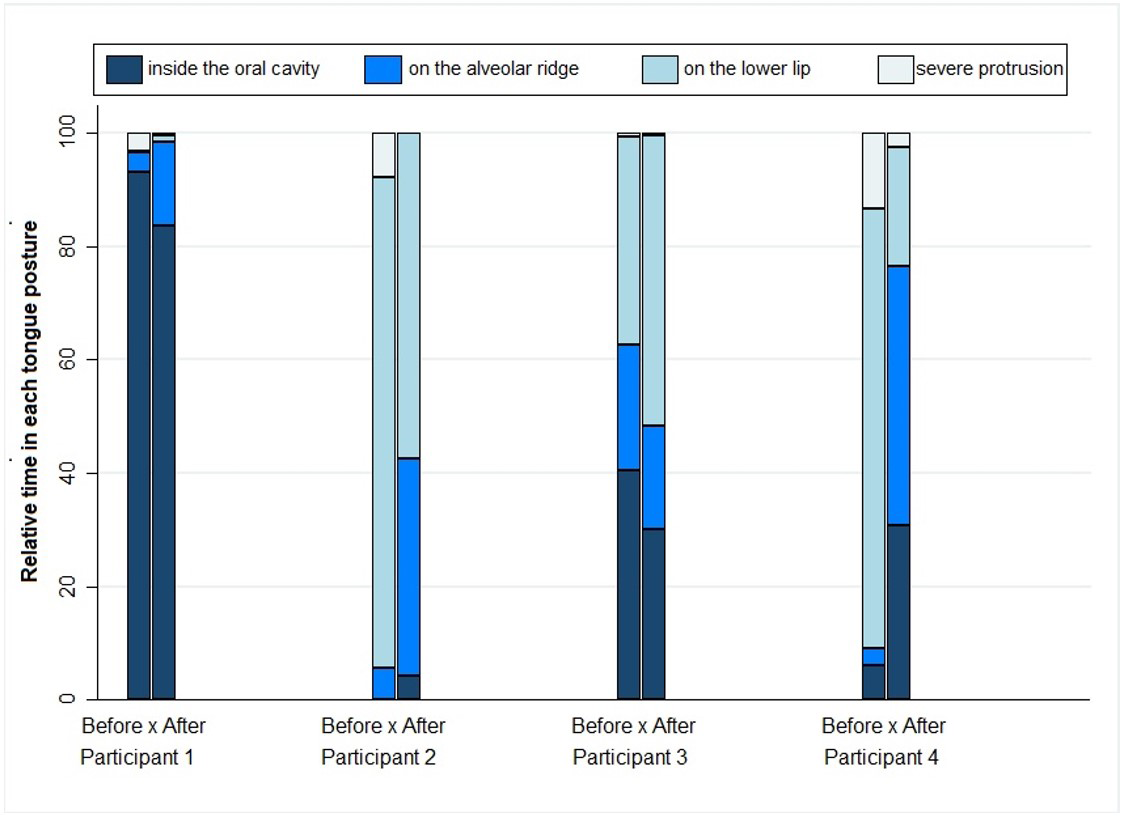
Percentage of the time participants remained in each tongue posture at the beginning and end of the treatment in relation to video duration

Figures 2 and 3 present the percentage of the time each participant remained in the various tongue and lip postures in the video recordings made at the beginning and end of the treatment in relation to the total useful time of the videos - i.e., excluding the moments when they smiled, cried, or vocalized.

The comparison of tongue and lip posture at the beginning and end of the treatment shows that all participants decreased their time in severe tongue protrusion and in relation to the lower lip. Also, all of them except for participant 1 increased their time with closed lips.

Participant 1 remained longer with open lips at the end of the treatment but importantly decreased the severe tongue protrusion in relation to the lower lip. Participant 2 decreased by 20% the time with open lips and no longer had severe tongue protrusion in relation to the lower lip by the end of the treatment. Participant 3 decreased by almost 10% the time with open lips but started positioning the tongue on the lower lip for longer. Participant 4 decreased by almost 69% the time with open lips and started positioning the tongue between the alveolar ridges at the end of the treatment.

Concerning functional aspects in the reassessment at the end of the treatment, none of the children had deleterious sucking habits, and the families had no complaints of choking.

## DISCUSSION

Muscle hypotonia, which is characteristic of individuals with T21, impairs orofacial development, causing functional limitations in sucking, breathing, mastication, and speech^(3)^. Previous studies have already used video recordings to assess the effects of SPP treatment in children with T21^(8,11)^ and indicated the advantages of this method in comparison with photographs, clinical observation alone, and parental reports.

In the present study, participant 1, who began the treatment at 6 months old, remained less time with closed lips at the end of the treatment and started keeping them predominantly open instead. This participant also spent less time with the tongue inside the oral cavity at the end of the treatment. Despite these two negative findings, the severe tongue protrusion in relation to the lower lip decreased. It must be pointed out that case 1 had better postural conditions of the speech articulation organs than all other participants, and that the literature indicates that the best results of this therapeutic approach occur in the most severe cases^(13)^.

Participant 2 began the treatment at only 1 month old. It is believed that beginning the treatment early, in this case, was responsible for the good treatment results. In the end, the participant no longer had severe tongue protrusion in relation to the lower lip and decreased open lips considerably, with the best results of the four cases analyzed. According to Castillo-Morales^(5)^, this therapy is more effective when conducted as early as possible, preferably during the period of greater development of the oral cavity and central nervous system. Furthermore, by 6 months old the child can already lateralize the tongue and make protrusion movements more often to expel it from their oral cavity^(14)^, diminishing the time of plate use. Teeth eruption is also considered a barrier to SPP retention^(15)^.

Even though studies point out greater benefits when it begins early^(15)^, positive results were also reported in older children^(6)^. A clinical case study that used this therapeutic approach in a child 3 years and 10 months old diagnosed with T21 found that the subject improved lip closure and tongue posture after 4 months of treatment^(6)^. This shows that children older than 1 year can also benefit from the treatment. Contrary to these authors and corroborating those who favor an early treatment^(5,15)^, participant 3, who began the therapy at 18 months old, started positioning the tongue on the lower lip for longer. This case improved only in lip posture.

Participant 4, who began the therapy at 2 months old, had habitual open lips 93% of the time at the beginning of the treatment and started having parted lips at the end of it. Moreover, they started positioning the tongue between the alveolar ridges for most of the time at the end of the therapy - which is an improvement in this aspect, as their predominant habitual tongue posture at the beginning of it was on the lower lip. This indicates that the treatment improved the patient’s muscles and reinforces that positive results are found when the treatment begins early^(16)^. Regarding tongue posture, this patient had the greatest severity of all four participants in the initial assessment (severe protrusion 13.4% of the time and protrusion on the lower lip 77.7% of the time) and kept their lips open 93.8% of the time. This finding, associated with participant 2’s positive results (who likewise had poor postural conditions at the beginning of the treatment), agrees with the literature, which indicates better results in cases with more changes in the initial assessment^(13)^.

It is important to emphasize that the age at the beginning of the treatment and the severity of postural changes in speech articulation organs were not the only variables that differed between participants. Other non-investigated aspects, such as adherence to the treatment, were not controlled, which poses a research bias.

A literature review on early therapy for orofacial changes in children with T21 pointed out that because of the wide range of elements that make up SPP and ORT treatment, is it impossible to ascribe specific effects to its various components. Hence, SPP treatment alone, without ORT, is not recommended. Another gap in the published studies concerning therapy with SPP and ORT is that none of the articles that were found clearly described which exercises were used in ORT^(17)^.

This is a case report study and, therefore, its results cannot be generalized, and the conclusions refer specifically to the study cases. Limitations of the study include the subjectivity of the habitual tongue and lip posture assessment. Also, 5-minute recordings may not reliably represent the habitual posture children used at other times of the day. A strength of the study was that the assessments were recorded and analyzed by two researchers, which increased the reliability of the results. Further studies should address SPP therapy associated with orofacial myofunctional therapy in larger samples and assess them months after ending the treatment to verify whether the results remain. They should also control adherence to SPP use and orofacial myofunctional therapy.

## FINAL COMMENTS

It was found that SPP associated with myofunctional therapy had better tongue and lip habitual posture results in patients who began the therapy at 1 and 2 months old and had poorer postural conditions in the initial assessment.
