# Supplementary material for: Orofacial myofunctional therapy associated with the use of the stimulating palatal plate in children with trisomy 21: case studies
Source: Codas. 2023 Sep 1;35(5):e20210231. doi: 10.1590/2317-1782/20232021231en (PMC10546989; doi:10.1590/2317-1782/20232021231en)
Supplement: Translation

# Translation: Terapia miofuncional orofacial associada ao uso da placa palatina de memória em crianças com Trissomia do 21: estudo de casos

**DOI:** 10.1590/2317-1782/20232021231pt

**Authors:** Jéssica Ellen de Almeida Ferreira, Bruna Rezende Santos de Almeida, Tahyná Duda Deps, Henrique Pretti, Renata Maria Moreira Moraes Furlan

**Affiliations:** 1 Graduação em Fonoaudiologia, Faculdade de Medicina, Universidade Federal de Minas Gerais - UFMG - Belo Horizonte (MG), Brasil.; 2 Departamento de Fonoaudiologia, Faculdade de Medicina, Universidade Federal de Minas Gerais - UFMG - Belo Horizonte (MG), Brasil.; 3 Faculdade de Tecnologia do Ipê - FAIPE - Cuiabá (MT), Brasil.; 4 Departamento de Dentística Restauradora, Faculdade de Odontologia, Universidade Federal de Minas Gerais - UFMG - Belo Horizonte (MG), Brasil.

**Keywords:** Síndrome de Down, Hipotonia Muscular, Reabilitação, Terapia Miofuncional, Aparelhos Ortopédicos

## Abstract

Indivíduos com Trissomia do 21 podem apresentar hipotonia muscular dos órgãos fonoarticulatórios, língua alargada, posicionada no assoalho oral e protrusa e ausência de selamento labial. A placa palatina de memória é um dispositivo intraoral que, associado à terapia miofuncional, visa à melhora da postura habitual dos lábios e da língua dessas crianças. O objetivo deste trabalho foi apresentar os casos de quatro crianças com Trissomia do 21, do sexo masculino, com média de idade de 6,7 e desvio-padrão de 7,8 meses, que fizeram uso da placa palatina de memória de forma associada à terapia miofuncional. As crianças utilizaram a placa por seis meses, realizaram exercícios baseados na terapia de regulação orofacial e receberam orientações quanto à alimentação e retirada de hábitos orais deletérios. Na primeira sessão e ao final do tratamento, foi realizada a gravação de 5 minutos da face de cada criança em repouso e a análise da postura habitual de língua e de lábios foi realizada por dois pesquisadores independentes. Observou-se maior melhora da postura de língua e de lábios dos participantes que iniciaram o tratamento mais precocemente e que apresentavam as alterações posturais mais severas.

## INTRODUÇÃO

A Trissomia do 21 (T21) é uma alteração cromossômica caracterizada por uma série de modificações congênitas que interferem no desenvolvimento motor e neurofisiológico, tais como disfunções motoras e hipotonia muscular^(1)^. Segundo dados do Ministério da Saúde^(2)^, um a cada 700 nascidos no Brasil apresenta diagnóstico de T21, totalizando cerca de 270 mil pessoas. Indivíduos com T21 podem apresentar a maxila com tamanho diminuído, hipoplasia do terço médio da face, protrusão de língua e dificuldade no fechamento dos lábios^(3)^. Essas alterações têm impacto direto em funções como mastigação, deglutição, fonação e respiração^(4)^.

Durante a década de 70, o médico argentino Castillo-Morales^(5)^ desenvolveu um método de reabilitação neuromotora para crianças deficientes que consiste na Terapia de Regulação Orofacial (TRO), realizada por meio de exercícios para estimulação da musculatura. Associado à TRO, ele propunha o uso de um aparelho intraoral, a Placa Palatina de Memória (PPM).

A PPM é um aparelho confeccionado por dentistas a partir de moldagem do arco superior da criança, descrito em estudos sobre tratamento de indivíduos com T21, que visa promover adequação da posição habitual de língua e viabilizar o fechamento de boca^(6,7)^. Um estudo longitudinal que acompanhou vinte crianças com T21 em uso de PPM, associada à TRO, durante quatro anos, apontou que o tratamento gerou efeito positivo na função motora oral, principalmente no primeiro ano de vida das crianças, destacando-se melhorias no tônus e vedamento labial^(8)^.

Existem poucos estudos a respeito dos benefícios do uso da PPM associada à terapia miofuncional, e o tempo de tratamento para que as mudanças musculares ocorram ainda não está bem definido. Sendo assim, este trabalho relata os resultados do uso da placa palatina de memória, associada à terapia miofuncional, na postura de língua e de lábios, durante seis meses, em crianças com Trissomia do 21.

## APRESENTAÇÃO DOS CASOS CLÍNICOS

Um estudo de quatro casos, de natureza prospectiva, foi realizado. A pesquisa foi aprovada pelo Comitê de Ética em Pesquisa da instituição (CAAE 37828920.1.0000.5149 - Número do Parecer: 4.381.966). Os pais ou responsáveis legais dos participantes assinaram o Termo de Consentimento Livre Esclarecido, consentindo a realização da pesquisa e divulgação de seus resultados.

Os participantes da pesquisa consistiram de quatro crianças com diagnóstico de T21, todas do sexo masculino, com média de idade de 6,7 meses e desvio-padrão de 7,8 meses. Nenhuma delas apresentava outras síndromes associadas, más-formações craniofaciais, distúrbios cardíacos ou respiratórios. Tais participantes foram recrutados dentre aqueles encaminhados para atendimento em um projeto de extensão da Universidade Federal de Minas Gerais.

As crianças foram avaliadas por dentista e fonoaudiólogo na primeira sessão. A avaliação miofuncional orofacial compreendeu a avaliação do tônus^(9,10)^, da postura habitual de lábios e de língua^(11)^, do frênulo lingual^(10)^, bem como a investigação sobre a alimentação^(9)^ e presença de hábitos orais^(10)^. Todas apresentaram lábios e língua com tônus diminuído e postura habitual alterada e frênulo de língua sem alterações. Os principais achados orofaciais miofuncionais encontram-se descritos no Quadro 1.

**Quadro 1:** Principais achados da avaliação miofuncional orofacial das crianças

**Participante (idade)**	**Principais achados da avaliação miofuncional**
Participante 1 (6 meses)	**Tônus de lábios e de língua:** diminuído;
	**Postura de língua:** no assoalho oral;
	**Postura de lábios**: ora abertos ora fechados;
	**Alimentação**: líquido em mamadeira e papa de fruta ofertada em colher de plástico de tamanho pequeno, sem queixas. Amamentação até 3 meses de idade;
	**Hábitos deletérios de sucção**: ausentes;
	**Dentes:** ausentes.
Participante 2 (um mês)	**Tônus de lábios e de língua:** diminuído;
	**Postura de língua:** sobre o lábio inferior;
	**Postura de lábios:** abertos;
	**Alimentação:** aleitamento materno complementado com fórmula, sem queixas;
	**Hábitos deletérios de sucção**: ausentes;
	**Dentes**: ausentes.
Participante 3 (18 meses)	**Tônus de lábios e de língua:** diminuído;
	**Postura de língua:** intercalando entre sobre o lábio inferior e dentro da cavidade oral, porém, rebaixada no assoalho oral;
	**Postura de lábios:** abertos;
	**Alimentação:** sólidos, líquidos e pastosos, com queixa de engasgos esporádicos para sólidos;
	**Hábitos deletérios de sucção:** ausentes;
	**Dentes:** presença dos incisivos centrais superiores e inferiores.
Participante 4 (2 meses)	**Tônus de lábios e de língua:** diminuído;
	**Postura de língua:** sobre o lábio inferior;
	**Postura de lábios:** abertos;
	**Alimentação:** aleitamento materno complementado com fórmula, queixas de engasgos;
	**Hábitos deletérios de sucção:** sucção de chupeta;
	**Dentes:** ausentes.

O dentista realizou o molde do arco superior de cada participante para confecção da PPM, que foi entregue, na semana seguinte, aos responsáveis pela criança. Um exemplo de PPM pode ser observado na Figura 1.

**Figura 1:** Placa Palatina de Memória (PPM)

Ainda na primeira sessão, o fonoaudiólogo realizou gravação de vídeo da face da criança com duração de 5 minutos. A gravação foi realizada com máquina digital semiprofissional da Marca Sony, modelo DSC-H50 (Sony^®^, Manaus, Brasil) em alta qualidade. A criança foi posicionada no bebê conforto ou no colo do responsável, o qual foi orientado a não interferir nas gravações. No momento da gravação, a criança não estava fazendo uso da PPM. Brinquedos apropriados a cada idade foram oferecidos para manter a criança distraída, uma vez que o intuito foi captar a postura habitual de lábios e língua.

Na segunda sessão, que ocorreu após uma semana da avaliação, os participantes receberam a PPM e os pais foram orientados a inseri-la na cavidade oral da criança, quatro vezes ao dia, pelo período mínimo de 30 minutos^(12)^. Também foram orientados quanto aos procedimentos de higienização da placa e para que não a utilizassem no momento das refeições ou durante o sono^(5)^. Além disso, foram solicitados a realizar, diariamente, algumas estratégias terapêuticas visando ao fortalecimento da musculatura orofacial, as quais se encontram descritas no Quadro 2 ^(5)^. Todas estas estratégias foram realizadas pela fonoaudióloga responsável pela pesquisa, na presença dos pais, os quais foram treinados para repeti-las em casa, bem como foram filmadas e os vídeos disponibilizados à família, juntamente com uma cartilha contendo explicações sobre as mesmas.

**Quadro 2:** Exercícios indicados no tratamento

**Exercício**	**Descrição**	**Repetição**
**1) Fortalecimento do masseter**	Com as pontas dos dedos de ambas as mãos, deslizar no sentido de baixo para cima, do ângulo da mandíbula em direção ao olho.	10 vezes cada movimento
**2) Alongamento de lábio superior**	Com as pontas dos dedos indicador e polegar, deslizar do centro do lábio superior para a margem e de cima para baixo.	10 vezes cada movimento
**3) Alongamento de lábio inferior**	Com as pontas dos dedos indicador e polegar, deslizar do centro do lábio inferior para a margem e de baixo para cima.	10 vezes cada movimento
**4) Estimulação da zona motora nasal inferior**	Com o dedo indicador horizontalmente sobre o lábio superior, realizar pressão e vibração para cima e para trás.	10 vezes cada movimento
**5) Estimulação da zona motora dos lábios**	Com as pontas dos dedos indicadores sobre o músculo zigomático maior, pressionar, vibrar e puxar ao mesmo tempo.	10 vezes cada movimento
**6) Estimulação da zona motora do mento**	Com o dedo indicador sob a face, posicionar o dedo polegar no queixo, e fazer movimento para baixo junto com pressão e vibração.	10 vezes cada movimento
**7) Estimulação da zona motora da língua**	Com o polegar ou indicador abaixo da face, na região submandibular do pescoço, realizar pressão e vibração intermitente.	10 vezes cada movimento
**8) Vibração de língua**	Colocar o dedo indicador sobre a língua e realizar pressão e vibração intermitente. Logo em seguida, fazer o controle da mandíbula.	20 vezes cada movimento
**9) Elevação do ápice da língua**	Com a ponta do dedo indicador atrás da gengiva inferior, sem tocar os dentes, e o dedo polegar na região submandibular, erguer a ponta do dedo indicador para levar a língua para perto dos dentes superiores. Logo em seguida, fazer o controle da mandíbula.	10 vezes cada movimento
**10) Afilamento de língua**	Com o indicador, ou com uma escova de dentes, tocar a margem lateral da língua no sentido de frente para trás.	10x de cada lado da língua
**11) Vibração dos lábios**	Flexionar as articulações dos dedos formando uma concha com as mãos. Colocar a mão sobre os lábios da criança, com cuidado para não impedir a respiração pelo nariz. Fazer uma pressão sobre os lábios e face, produzindo um efeito de vácuo. Em seguida vibrar no sentido de baixo para cima.	20 vezes cada movimento
**12) Controle de mandíbula**	Posiciona-se o dedo indicador no queixo, o dedo médio abaixo da mandíbula e o polegar ao longo do contorno da mandíbula. O braço apoia a cabeça.	Sempre que for necessário alinhar a criança.

Fonte: Castillo-Morales^(5)^.

A terceira sessão foi realizada após 14 dias da segunda e a quarta após dois meses. Tais sessões tiveram por objetivo reforçar as orientações relacionadas às estratégias terapêuticas visando ao fortalecimento da musculatura orofacial. Além disso, foram realizadas orientações às famílias para retirada de hábitos orais deletérios, sobre pega correta e posicionamento durante a amamentação para os casos 2 e 4 e sobre o modo de oferta dos alimentos, posicionamento da criança durante a oferta e utensílios apropriados para os casos 1 e 3. Essas sessões foram realizadas na modalidade presencial.

Os profissionais ficaram à disposição das famílias para sanar dúvidas relacionadas ao uso da placa e aos exercícios por telefone ou por teleconsulta sempre que eles julgassem necessário. O número de sessões, para cada caso clínico variou conforme a necessidade de confecção da nova placa ou de dúvidas apresentadas pelas famílias. Os casos 1 e 3 realizaram quatro sessões presenciais e uma sessão por teleatendimento. Os casos 2 e 4 realizaram seis sessões presenciais e uma sessão por teleatendimento, pois a PPM precisou ser refeita devido ao crescimento do palato da criança, daí o aumento de uma sessão para moldagem e outra para recebimento da nova placa.

Após seis meses de tratamento, foi realizada reavaliação e nova gravação da face, mantendo condições similares às da avaliação inicial. No momento das gravações, a criança não estava fazendo uso da PPM.

Os vídeos foram analisados, quadro a quadro, por duas pesquisadoras de forma independente. Em cada quadro, a postura de língua da criança foi classificada como: I) dentro da cavidade oral (língua atrás do rolete gengival inferior ou atrás dos dentes incisivos inferiores); II) entre os roletes gengivais (língua sobre o rolete gengival inferior e atrás do lábio inferior); III) sobre o lábio inferior (língua tocando o lábio inferior); IV) protrusão severa em relação ao lábio inferior (língua protruída, sobre o lábio inferior, com o ápice ultrapassando o limite anterior do lábio inferior)^(11)^. A postura de lábios foi classificada como: I) fechados (presença de contato entre os lábios inferior e superior em toda a extensão da rima labial); II) semiabertos (contato entre os lábios superior e inferior apenas próximo às comissuras labiais); ou III) abertos (não há contato entre lábio inferior e superior). Foram contabilizados os segundos em que a criança permaneceu em cada classificação da postura habitual de lábios e de língua. Sendo que, os momentos em que a criança sorriu ou vocalizou não foram considerados na análise. Os dados foram comparados entre os momentos da avaliação e da reavaliação após 6 meses.

Para aumentar a confiabilidade dos dados, dois pesquisadores realizaram a análise dos vídeos. Para verificação da concordância entre eles, foi calculado o coeficiente de correlação de Spearman (Rho). Os dados de cada participante foram analisados de forma qualitativa. O coeficiente de correlação intraclasse (ICC) foi de 0,98 para o participante 1, 0,95 para o participante 2, 0,99 para o participante 3 e 0,98 para o participante 4, revelando excelente concordância interexaminador.

As Tabelas 1 e 2 apresentam o tempo de permanência de cada participante em diferentes posturas habituais de língua e de lábios nos vídeos realizados no início e ao final do tratamento.

**Tabela 1:** Tempo de permanência dos participantes em cada postura de lábios no início e final do tratamento

Momento da avaliação	Participante	Idade (meses)	Postura de lábios					
			Fechados		Semiabertos		Abertos	
			Tempo (min)	%	Tempo (min)	%	Tempo (min)	%
Início do tratamento	1	6	191	66,1	37	12,8	61	21,1
	2	1	1	0,4	2,5	0,9	269	98,7
	3	18	9,5	4,3	7,5	3,4	205	92,3
	4	2	8	3,3	7	2,9	226,5	93,8
Após 6 meses de tratamento	1	12	37	16,7	78	35,3	106	48,0
	2	7	1	0,7	28,5	20,6	109	78,7
	3	24	10	5,7	19,5	11,1	146	83,2
	4	8	9,5	8,2	73	62,7	34	29,2

**Legenda:** min = minutos.

**Tabela 2:** Tempo de permanência dos participantes em cada postura de língua no início e final do tratamento

Momento da avaliação	Participante	Idade (meses)	**Postura de língua**							
			Dentro da cavidade oral		Entre os roletes gengivais		Sobre o lábio inferior		Protrusão severa	
			Tempo (min)	%	Tempo (min)	%	Tempo (min)	%	Tempo (min)	%
Início do tratamento	1	6	271,5	93,3	9,5	3,3	0,5	0,2	9,5	3,3
	2	1	0	0,0	14	5,5	218,5	86,5	20	7,9
	3	18	87	40,4	48	22,3	79	36,7	1,5	0,7
	4	2	14,5	6,1	7	2,9	186	77,7	32	13,4
Final do tratamento	1	12	168	83,6	29,5	14,7	2,5	1,2	1	0,5
	2	7	7,5	4,2	68	38,3	102	57,5	0	0
	3	24	55,5	30	34	18,4	94,5	51,1	1	0,5
	4	8	36	30,8	53,5	45,7	24,5	20,9	3	2,6

**Legenda:** min = minutos.

As Figuras 2 e 3 indicam o tempo de permanência de cada participante nas diferentes posturas habituais de língua e de lábios nos vídeos realizados no início e ao final do tratamento, em porcentagem do tempo total útil de vídeo, ou seja, excluídos os momentos caracterizados como sorriso, choro ou vocalização.

**Figura 2:** Tempo de permanência dos participantes, em porcentagem do tempo de duração do vídeo, para cada postura de lábios no início e final do tratamento

**Figura 3:** Tempo de permanência dos participantes em porcentagem do tempo de duração do vídeo, para cada postura de língua no início e final do tratamento

Ao se comparar a postura de língua e de lábios no início e ao final do tratamento, todos os participantes apresentaram diminuição no tempo de postura de língua classificada como protrusão severa em relação ao lábio inferior. Além disso, todos, com exceção do participante 1, apresentaram aumento no tempo de permanência da postura de lábios fechados.

O participante 1 apresentou maior tempo de permanência de lábios abertos ao final do período de tratamento, porém apresentou diminuição importante na postura de língua com protrusão severa em relação ao lábio inferior. O participante 2 diminuiu em 20% o tempo de permanência de lábios abertos e, ao final do tratamento, não apresentou mais a postura de língua classificada como protrusão severa em relação ao lábio inferior. O participante 3 diminuiu em quase 10% o tempo de permanência de lábios abertos, porém passou a adotar a postura de língua sobre o lábio inferior por mais tempo. O participante 4 diminuiu em quase 69% o tempo de permanência de lábios abertos, e, ao final do tratamento, passou a adotar a postura de língua entre os roletes gengivais.

Com relação aos aspectos funcionais, na reavaliação realizada ao final do tratamento, nenhuma criança possuía hábitos deletérios de sucção e as famílias não apresentavam queixas de engasgos.

## DISCUSSÃO

A hipotonia muscular, característica de indivíduos com T21, prejudica o desenvolvimento orofacial, acarretando limitações funcionais na sucção, respiração, mastigação e fala^(3)^. Estudos prévios já utilizaram registros de vídeo para avaliar os efeitos do tratamento com a placa palatina de memória em crianças com T21^(8,11)^ e indicaram as vantagens desse método em comparação ao registro fotográfico, observação clínica isolada e relato dos pais.

Neste estudo, o participante 1, que iniciou o tratamento aos seis meses de idade, apresentou menor tempo de permanência de lábios fechados ao final do período de tratamento, passando a adotar predominantemente a postura de lábios abertos. Este participante também apresentou menor tempo de língua dentro da cavidade oral, ao final do tratamento. Apesar destes dois achados negativos, houve diminuição na postura de língua com protrusão severa em relação ao lábio inferior. Destaca-se o fato de que as condições posturais dos órgãos fonoarticulatórios do caso 1 eram as melhores dentre todos os participantes e que a literatura aponta que os melhores resultados para essa abordagem terapêutica são vistos nos casos de maior severidade^(13)^.

O participante 2 iniciou o tratamento com apenas um mês de vida. Acredita-se que o início precoce do tratamento neste caso foi responsável pelos bons resultados obtidos com o tratamento. Ao final, o participante não apresentou mais a postura de língua classificada como protrusão severa em relação ao lábio inferior e diminuiu consideravelmente a postura de lábios abertos, tendo apresentado os melhores resultados dentre os quatro casos analisados. Segundo Castillo-Morales^(5)^, a terapia é mais efetiva quando realizada o mais precocemente possível, de preferência, aproveitando o período de maior desenvolvimento da cavidade oral e do sistema nervoso central. Além disso, aos 6 meses a criança já é capaz de lateralizar a língua e realizar, mais frequentemente, movimentos de protrusão para expulsá-la da cavidade oral^(14)^, o que diminuiu o tempo de uso da placa. A erupção dentária também é considerada um dificultador para a retenção da PPM^(15)^.

Embora os estudos apontem para melhores benefícios se iniciado precocemente^(15)^, resultados positivos também foram relatados em casos com crianças mais velhas^(6)^. No estudo de um caso clínico em que essa abordagem terapêutica foi oferecida a uma criança de três anos e dez meses com diagnóstico de T21, foi observado que, após quatro meses de tratamento, a criança apresentou melhora no selamento labial e na postura de língua^(6)^. Isso demonstra que crianças com mais de um ano de idade também podem ter benefícios com o tratamento. Contrariando tais autores e corroborando aqueles a favor do tratamento precoce^(5,15)^, o participante 3, que iniciou o tratamento com dezoito meses, passou a adotar a postura de língua sobre o lábio inferior por mais tempo. A melhora neste caso foi vista apenas na postura de lábios.

O participante 4, que iniciou a terapia com dois meses de idade, apresentou a postura habitual de lábios abertos em 93% do tempo no início do tratamento, passando a adotar, ao final do tratamento, a postura de lábios semiabertos. Além disso, ao final do tratamento, passou a permanecer com a postura de língua entre os roletes gengivais na maior parte do tempo, o que representa melhora neste aspecto, visto que, no início, sua postura habitual de língua predominante era sobre o lábio inferior. Isso indica que o tratamento promoveu melhora na musculatura do paciente e reforça o fato de que resultados positivos são encontrados quando o início do tratamento acontece de forma precoce^(16)^. Este paciente, no que se refere à postura de língua, apresentava na avaliação inicial a maior severidade dentre os quatro participantes (protrusão severa em 13,4% do tempo e protrusão sobre o lábio inferior em 77,7% do tempo), além de permanecer com lábios abertos em 93,8% do tempo. Este achado, associado aos resultados positivos apresentados pelo participante 2, que também apresentava condições posturais ruins no início do tratamento, condiz com a literatura que aponta para os melhores resultados nos casos com maiores alterações na avaliação inicial^(13)^.

Vale ressaltar que a idade de início do tratamento e a gravidade das alterações posturais dos órgãos fonoarticulatórios não foram as únicas variáveis que se diferiam entre os participantes. Outros aspectos, não investigados, como a adesão ao tratamento não foram controlados, o que constitui um viés da pesquisa.

Uma revisão da literatura sobre a terapia precoce para as alterações orofaciais de crianças com T21 apontou que, por causa da grande variedade de elementos que fazem parte do tratamento com uso da PPM e TRO, é impossível atribuir os efeitos específicos para os diferentes componentes que o constituem. Por isso, o tratamento utilizando a PPM isoladamente, sem realizar a TRO não é recomendado. Outra lacuna nos estudos já publicados a respeito da terapia com a PPM e a TRO, é que nenhum dos artigos encontrados descreveu com clareza quais foram os exercícios usados na TRO^(17)^.

O presente estudo constitui um relato de casos, por isso, os resultados obtidos impossibilitaram a generalização e as conclusões são específicas para os casos estudados. Limitações do estudo referem-se à subjetividade da avaliação da postura habitual de língua e de lábios e ao fato de que uma gravação de cinco minutos pode não representar fidedignamente a postura habitual adotada por estas crianças em outros momentos do dia. Como ponto forte do estudo tem-se o fato de que as avaliações foram gravadas e analisadas por dois pesquisadores, o que aumentou a confiabilidade dos resultados. Sugerem-se outros estudos sobre a terapia com o uso da Placa Palatina de Memória associada à terapia miofuncional orofacial com amostras maiores e com avaliação realizada meses após o encerramento do tratamento, a fim de se verificar se os resultados permanecem. Além disso, que os futuros estudos controlem a adesão ao uso da Placa Palatina de Memória e à terapia miofuncional orofacial.

## COMENTÁRIOS FINAIS

Foi possível observar que a Placa Palatina de Memória associada à terapia miofuncional teve melhores resultados para a postura habitual de língua e lábios dos pacientes que iniciaram a terapia com um e dois meses de idade e apresentavam condições posturais mais alteradas na avaliação inicial.
